# Investigation of aquaporins and apparent diffusion coefficient from ultra-high b-values in a rat model of diabetic nephropathy

**DOI:** 10.1186/s41747-017-0016-3

**Published:** 2017-10-10

**Authors:** Yu Wang, Heng Zhang, Ruzhi Zhang, Zhoushe Zhao, Ziqian Xu, Lei Wang, Rongbo Liu, Fabao Gao

**Affiliations:** 1 Department of Radiology, West China Hospital of Sichuan University, No. 37, Guoxue Lane outside the south, Wuhou District Chengdu, China; 2 General Electronic Company Healthcare (China), Beijing, China

**Keywords:** Diffusion-weighted imaging (DWI), Diabetic nephropathy, Aquaporins, Apparent diffusion coefficient (ADC), Ultra-high b-values

## Abstract

**Background:**

To assess kidney damage in a rat model of type-2 diabetic nephropathy based on apparent diffusion coefficient (ADC) data obtained from ultra-high b-values and discuss its relationship to the expression of aquaporins (AQPs).

**Methods:**

This study was approved by the institutional Animal Care and Use Committee. Thirty male Sprague-Dawley rats were randomised into two groups: (1) untreated controls and (2) diabetes mellitus (DM). All rats underwent diffusion-weighted imaging (DWI) with 18 b-values (0–4500 s/mm^2^). Maps of low ADC (ADC_low_), standard ADC (ADC_st_) and ultra-high ADC (ADC_uh_) were calculated from low b-values (0–200 s/mm^2^), standard b-values (300–1500 s/mm^2^) and ultra-high b-values (1700–4500 s/mm^2^), respectively. The expression of AQPs in the kidneys was studied using immunohistochemistry. Laboratory parameters of diabetic and kidney functions, ADC_low_, ADC_st_, ADC_uh_, and the optical density (OD) of AQP expression in the two groups were compared using an independent *t* test. Correlations between ADCs and the OD of AQP expression were evaluated by Pearson’s correlation analysis.

**Results:**

ADC_uh_ were significantly higher in the cortex (CO), outer stripe of the outer medulla (OS) and inner stripe of the outer medulla (IS), and the OD values of AQ-2 were significantly higher in the OS, IS and inner medulla (IM) in DM animals compared with control animals. ADC_uh_ and OD values of AQP-2 expression were positively correlated in the OS, IS and IM of the kidney.

**Conclusions:**

ADC_uh_ may work as useful metrics for early detection of kidney damage in diabetic nephropathy and may be associated with AQP-2 expression.

## Keypoints

- Diffusion-weighted imaging (DWI) with ultra-high b-values may be valuable for the detection of diabetic nephropathy
- Apparent diffusion coefficient (ADC) calculated using ultra-high b-values is different between diabetes mellitus (DM) and control animals
- ADC_uh_ may be associated with water transportation by aquaporins

## Background

As a global health problem that affects multiple organs of the body, diabetes mellitus (DM) is known to cause proteinuria, nephrotic syndrome in the kidney, and may ultimately result in renal failure [1]. A high urinary glucose level induces elevated urinary osmotic pressure, which in turn reduces renal water reabsorption, and finally leads to polyuria [2]. Although persistent polyuria is reported to cause severe dehydration, shock from serious dehydration rarely develops in diabetic patients. It is, therefore, believed that the kidney has a compensating mechanism that alleviates diabetic dehydration. Water channels or aquaporins (AQPs) are membrane-associated proteins that regulate transcellular water movement across the cell membrane. So far, at least 13 mammalian AQPs (AQP 0–12) have been identified, eight of which are constitutively expressed in the kidney [3] and play a vital role in the reabsorption of water from the renal tubular fluid [4].

Several recent studies have used diffusion-weighted imaging (DWI) methods to evaluate renal function in diabetic nephropathy [5–7]. However, their values of the apparent diffusion coefficient (ADC) obtained from standard b-values (0, 1000 s/mm^2^) do not differentiate early DM patients from controls in a consistent manner. Through linear regression, images taken at various b-values can be used to calculate the ADC in a particular region of interest. However, ADC estimates are susceptible to the impact of physiological changes in vivo, such as tubular flow and vascular flow, because true water diffusion contributes mainly to the signal acquired with b-values larger than approximately 200 s/mm^2^ whereas the signal from blood perfusion with a rapid flow in tubular and vascular tubes disappears together with b-values greater than 200 s/mm^2^ [8]. Recently, researchers who have used ultra-high b-value-DWI in some diseases suggested that ADC, when calculated using ultra-high b-values (ADC_uh_), may help to reveal AQP expression [9]. Thus, the question is: Can the slower water diffusion through the AQPs be quantified by DWI?

Therefore, this study, was aimed at assessing kidney damage in DM to verify the association between ADC_uh_ and the permeability of AQPs in the cell membrane.

## Methods

### Animals and DM induction

All the experiments were conducted according to the guidelines of the Animal Care and Use Committee of Sichuan University, Chengdu, China and the Animal Ethics Committee Guidelines of the Animal Facility of the West China Hospital, Chengdu, China and had been given prior approval by the Experimental Animal Management Committee of Sichuan University, Chengdu, China under Contract 2016007A. Two groups of male Sprague-Dawley rats (Dashuo, Chengdu, China) were examined by magnetic resonance imaging (MRI): (1) untreated controls (*n* = 15) and (2) diabetic animals (*n* = 15). At the age of 6 to 7 weeks (weighing 180–200 g), the animals were fed a high-sucrose and high-fat diet (SCXK2014-028 Research Diet). Four weeks after the diet change, at the age of 10 to 11 weeks, DM was induced in group-2 animals by an intraperitoneal injection of a low dose of streptozotocin (STZ) (40 mg/kg Meilune). After the STZ treatment, 15 animals in the DM group that developed diabetes with a hyperglycemia of >16.7 mmol/lr underwent MRI.

### Physiological and laboratory parameters

General parameters, such as body weight and food and water intake, were monitored regularly. Forty days after diabetes induction, urine was collected over a period of 24 h and urinary albumin and creatinine concentrations were quantified. Blood was collected from the tail vein. Serum glucose, urea, albuminuria and creatinine were determined using a clinical analyser (cobas c311, Roche, Basel, Switzerland). Creatinine clearance was calculated from serum and urinary creatinine concentrations and the urine volume, which was collected over a period of 24 h.

### MRI protocol

Forty days after DM induction, all the animals underwent MRI examination using a 7.0-T small-animal scanner (Bruker Biospec 70/30, Ettlingen, Germany) and a rat heart array coil (Bruker Z114784). The animals were anaesthetised with 2% isoflurane/oxygen mixture throughout the MRI examination. The body temperature was kept constant at 37 °C using a heating blanket monitored with a rectal temperature probe. The respiration was monitored and kept constant at between 35 to 45 breaths per min throughout the examination. Respiratory motion was reduced by imaging the animals in the supine position and with respiratory trigger. Standard T2-weighted imaging was performed in axial and oblique coronal planes (repetition time/echo time 2032/27 ms; number of excitations 4; matrix 256 × 256; field of view 60 × 60 mm^2^; slice thickness 1.5 mm.) The coronal plane was orientated along the long axis of the kidney. To assess the diffusion, fat-saturated echo planar diffusion-map sequences were acquired (repetition time/echo time 800/30 ms; number of excitations 4; matrix 128 × 128; field of view 60 × 60 mm^2^; slice thickness 1.5 mm; three slices; b-values 0, 50,100, 150, 200, 300, 500, 800, 1000, 1300, 1500, 1700, 2000, 2500, 3000, 3500, 4000 and 4500 s/mm^2^). Scan times for the T2-weighted sequence and the diffusion-map sequence were about 4 min and 5 min, respectively. The total scan time of the whole examination was approximately 16 min.

### MRI data analysis

**Fig. 1:**
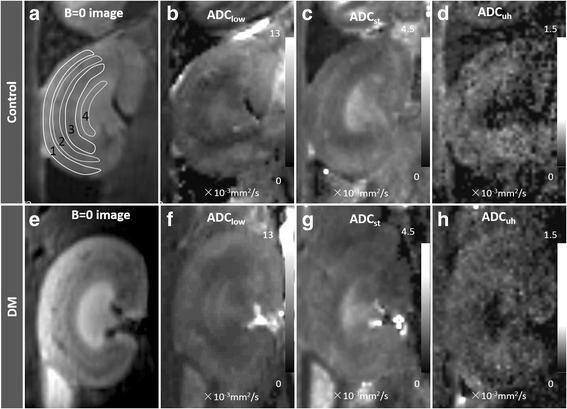
Example of diffusion maps in an animal with streptozotocin-induced diabetes mellitus (DM) and a control animal. **a-d**, b = 0 image, ADC_low_, ADC_st_, and ADC_uh_ maps in a DM animal. **e-h**, b = 0 image, ADC_low_, ADC_st_ and ADC_uh_ maps in a control animal. On b = 0 images, the four anatomical layers of the kidney can be distinctively observed. In these diffusion maps, regions of interest were placed onto the cortex (1), the outer stripe of the outer medulla (2), the inner stripe of the outer medulla (3) and the inner medulla (4) as exemplarily demonstrated in **a**. In DM animals, ADC_uh_ increased apparently in CO, OS and IS compared with controls (panels **d** and **h**)

Parameter maps of ADC were calculated using a monoexponential fit. The images were analysed by a radiologist blinded to the laboratory findings on an external workstation using Bruker software (ParaVision 5.0pl3 in/opt/PV5.0). The renal cortex (CO), the outer stripe of the outer medulla (OS), the inner stripe of the outer medulla (IS) and the inner medulla (IM) were identified as previously described [10]. On b = 0 images (which is essentially a T2-weighted image), regions of interest were placed onto the four anatomical layers of the kidney and copied to the ADC maps (Fig. 1). Maps of low ADC (ADC_low_), standard ADC (ADC_st_) and ADC_uh_ were calculated from low b-values (0–200 s/mm^2^), standard b-values (300–1500 s/mm^2^) and ultra-high b-values (1700–4500 s/mm^2^), respectively. Mean ADC values were determined respectively for each of the anatomical layers in all the animals.

### Renal immunohistochemistry

Kidney tissue samples (DM *n* = 15; controls *n* = 15) from the rats were obtained immediately after MRI. The rats were euthanised and underwent fixation via abdominal aortic perfusion with 4% paraformaldehyde in 1% phosphate-buffered saline. Coronal kidney sections (from 2 to 3 mm thick) were fixed overnight and embedded in paraffin. The paraffin sections were cut on a sliding microtome at 4 μm per section.

The sections were stained for AQP-1, AQP-2 and AQP-4 using immunohistochemical staining with anti-AQP-1 antibodies (ab15080, Abcam, Shanghai, China), anti-AQP-2 antibodies (ab15081, Abcam, Shanghai, China) and anti-AQP-4 antibodies (ab9512, Abcam, Shanghai, China), respectively. After accepting the antigen retrieved by using citrate antigen retrieval solution (P0081, Beyotime, Shanghai, China), the tissue sections were treated with 3% hydrogen peroxide to inactivate endogenous peroxidase. The sections were then incubated in a 1:20 goat serum for 30 min, rinsed, and incubated overnight at 4 °C with a 1:200 dilution of primary antibodies, including anti-AQP-1 antibodies, anti-AQP-2 antibodies and anti-AQP-4 antibodies. After three washes with phosphate-buffered saline, the sections were incubated for 90 min with biotinylated goat anti-rabbit immunoglobulin G antibody (SP-9001; Zhongshan Golden Bridge Biotechnology, Beijing, China). The sections underwent another three washes with phosphate-buffered saline and were then incubated with avidin-biotinylated horseradish peroxidase (SP-9001; Zhongshan Golden Bridge Biotechnology, Beijing, China) for 90 min. The sections were rewashed three times in phosphate-buffered saline and then incubated with diaminobenzidine and hydrogen peroxide (ZLI-9018; Zhongshan Golden Bridge Biotechnology, Beijing, China). The nucleus was stained with hematoxylin. The sections were then rinsed in water for 10 min, dehydrated and covered with a coverslip for microphotography.

High-powered images (×400 magnification) of the four anatomical layers of each kidney were taken separately (Leica ICC50 HD, Wetzlar, Germany). To analyse AQP-1, AQP-2 and AQP-4 staining, the regions covered by AQP-1, AQP-2 and AQP-4 staining were calculated by optical density (OD) [11]. We measured the OD values three times and averaged the results.

### Statistical analysis

Statistical analysis was performed with SPSS 19.0 (SPSS Inc., Chicago, IL, USA). Quantitative data were tested for normal distribution by using the Kolmogorov-Smirnov test. Normally distributed data were expressed as mean ± standard deviation. Laboratory parameters of diabetic and kidney functions, ADC_low_, ADC_st_, ADC_uh_, and the OD of AQPs expression in the two groups were evaluated with an independent *t* test with equal variance.. Correlation analysis between ADCs and OD values of AQPs of the anatomical layers was performed by Pearson’s correlation analysis. To avoid the introduction of type-I errors, Bonferroni’s correction was applied to the preset level of significance (*p* < 0.050). The significance levels were, therefore, as follows: *p* < 0.0083 (0.05/6) in Table 1; *p* < 0.017 (0.05/3) in Tables 2, 3 and 4.

**Table 1:** Characteristics of the two groups

	Controls	DM	*p* value
Body weight (g)	409 ± 23	286 ± 41	<0.001
Blood glucose (mmol/l)	5.8 ± 0.7	28 ± 4.1	<0.001
Creatinine clearance (ml/min)	1.5 ± 0.7	2.2 ± 1.1	0.048
Serum urea (mmol/l)	6.7 ± 0.6	9.7 ± 4.0	0.008
Urine output ml/24 h	9.5 ± 2.2	134 ± 21	<0.001
Urine albumin mg/l	2.5 ± 1.0	1.5 ± 0.7	0.007

Data are presented as mean ± standard deviation. Body weight and laboratory parameters were obtained after the day of the magnetic resonance imaging (MRI) scan. Comparisons between the diabetic group (DM) and controls were made by using an independent *t* test. Based on Bonferroni’s correction, *p* < 0.0083 (0.05/6) was considered to indicate a statistically significant difference

**Table 2:** Apparent diffusion coefficients (ADCs) in diabetes mellitus (DM) animals and controls

Position	Parameter (×10^-3^ s/mm^2^)	Controls	*DM*	*p* value
CO	ADC_low_	2.64 ± 0.525	2.52 ± 0.545	ns, 0.392
	ADC_st_	1.22 ± 0.230	1.30 ± 0.228	ns, 0.220
	ADC_uh_	0.196 ± 0.078	0.262 ± 0.050	<0.001
OS	ADC_low_	2.56 ± 0.543	2.36 ± 0.519	ns, 0.146
	ADC_st_	1.27 ± 0.226	1.31 ± 0.243	ns, 0.501
	ADC_uh_	0.190 ± 0.088	0.240 ± 0.067	0.016
IS	ADC_low_	2.33 ± 0.636	2.15 ± 0.536	ns, 0.250
	ADC_st_	1.29 ± 0.275	1.22 ± 0.218	ns, 0.316
	ADC_uh_	0.203 ± 0.081	0.268 ± 0.096	0.006
IM	ADC_low_	2.77 ± 0.561	2.66 ± 0.550	ns, 0.447
	ADC_st_	1.67 ± 0.305	1.60 ± 0.317	ns, 0.405
	ADC_uh_	0.140 ± 0.066	0.190 ± 0.101	ns, 0.027

Data are presented as mean ± standard deviation. Statistical analysis was performed using an independent *t* test between the two groups. *DM* diabetes mellitus, *CO* cortex, *OS* outer stripe of the outer medulla, *IS* inner stripe of the outer medulla, *IM* inner medulla, *ADC*_*low*_ ADC calculated using the low b-values, *ADC*_*st*_ ADC calculated using the standard b-values, *ADC*_*uh*_ ADC calculated using the ultra-high b-values, *ns* not significant. Based on Bonferroni’s correction, *p* < 0.017 (0.05/3) was considered to indicate a statistically significant difference

**Table 3:** Optical density of aquaporins (APQs) positively stained on different anatomical layers of the kidney in diabetes mellitus (DM) animals and controls

Position	AQP	Controls	DM	*p* value
CO	AQP-1	0.050 ± 0.013	0.049 ± 0.017	ns, 0.818
	AQP-2	0.026 ± 0.011	0.035 ± 0.010	ns, 0.025
	AQP-4	0.014 ± 0.006	0.012 ± 0.006	ns, 0.504
OS	AQP-1	0.056 ± 0.011	0.048 ± 0.014	ns, 0.095
	AQP-2	0.045 ± 0.011	0.060 ± 0.019	0.009
	AQP-4	0.011 ± 0.003	0.009 ± 0.004	ns, 0.234
IS	AQP-1	0.042 ± 0.010	0.039 ± 0.009	ns, 0.341
	AQP-2	0.036 ± 0.009	0.058 ± 0.016	0.000
	AQP-4	0.020 ± 0.005	0.019 ± 0.008	ns, 0.644
IM	AQP-1	0.036 ± 0.010	0.031 ± 0.009	ns, 0.140
	AQP-2	0.027 ± 0.010	0.040 ± 0.011	0.002
	AQP-4	0.026 ± 0.008	0.025 ± 0.009	ns, 0.694

Data are presented as mean ± standard deviation. Statistical analysis was performed using an independent *t* test between the two groups. *DM* diabetes mellitus, *DM* diabetes mellitus, *CO* cortex, *OS* outer stripe of the outer medulla, *IS* inner stripe of the outer medulla, *IM* inner medulla *ns*, not significant. Based on Bonferroni’s correction, *p* < 0.017 (0.05/3) was considered to indicate a statistically significant difference

**Table 4:** Correlation between apparent diffusion coefficients (ADCs) and aquaporin-2 (AQP-2) in diabetes mellitus (DM) animals at four anatomical layers of the kidney

Position	AQP	ADC_low_		ADC_st_		ADC_uh_	
		*r*	*p*	*r*	*p*	*r*	*p*
CO	AQP-1	−0.123	ns 0.509	0.026	ns 0.890	−0.023	ns, 0.903
	AQP-2	−0.178	ns 0.346	−0.044	ns 0.871	0.427	ns, 0.018
	AQP-4	−0.109	ns 0.565	−0.098	ns 0.607	−0.174	ns, 0.358
OS	AQP-1	0.123	ns 0.516	0.141	ns 0.458	−0.157	ns, 0.407
	AQP-2	−0.280	ns 0.134	−0.047	ns 0.806	0.598	0.000
	AQP-4	−0.193	ns 0.306	−0.139	ns 0.464	−0.194	ns, 0.304
IS	AQP-1	−0.266	ns 0.155	−0.106	ns 0.576	−0.026	ns, 0.893
	AQP-2	−0.277	ns 0.138	−0.139	ns 0.464	0.490	0.006
	AQP-4	−0.073	ns 0.700	−0.036	ns 0.850	0.140	ns, 0.460
IM	AQP-1	0.175	ns 0.355	0.293	ns 0.115	−0.039	ns, 0.838
	AQP-2	−0.109	ns 0.567	0.003	ns 0.986	0.451	0.012
	AQP-4	0.141	ns 0.459	0.173	ns 0.362	−0.227	ns, 0.227

The *r* and *p* values were obtained using Pearson’s correlation analysis. *DM* diabetes mellitus, *DM* diabetes mellitus, *CO* cortex, *OS* outer stripe of the outer medulla, *IS* inner stripe of the outer medulla, *IM* inner medulla *ns*, not significant. Based on Bonferroni’s correction, *p* < 0.017 (0.05/3 tests) was considered to indicate a statistically significant difference

## Results

### Physiological and laboratory parameters

Streptozotocin-induced DM reduced bodyweight gain but increased serum glucose in the DM animals compared with controls (*p* < 0.001; Table 1). In DM animals, creatinine clearance was no difference compared with controls (*p* = 0.048) but the urinary output of the diabetic group was higher than that of controls by 14-fold. In addition, serum urea elevated significantly in the DM group compared with controls (*p* = 0.008). Urine albumin levels of the DM group, however, were reduced in comparison with that of controls (*p* = 0.007; Table 1).

### DWI

All the MRI studies (*n* = 30) were included in the analysis. MRI could identify the four anatomical layers of the kidney (CO, OS, IS and IM) in all the animals (Fig. 1). An example of ADC_low_, ADC_st_ and ADC_uh_ maps in both a DM animal and a control animal is given in Fig. 1. ADC_uh_ of the CO, OS and IS in the DM animals was significantly higher than that in controls (Table 2). Although ADC_uh_ of the IM in the DM animals was significantly higher than that in controls at *p* < 0.050 level, there was no significant difference after Bonferroni’s correction. However, no significant difference was found in ADC_low_ and ADC_st_ in each region of interest between the two groups (Table 2).

### Immunohistochemistry

**Fig. 2:**
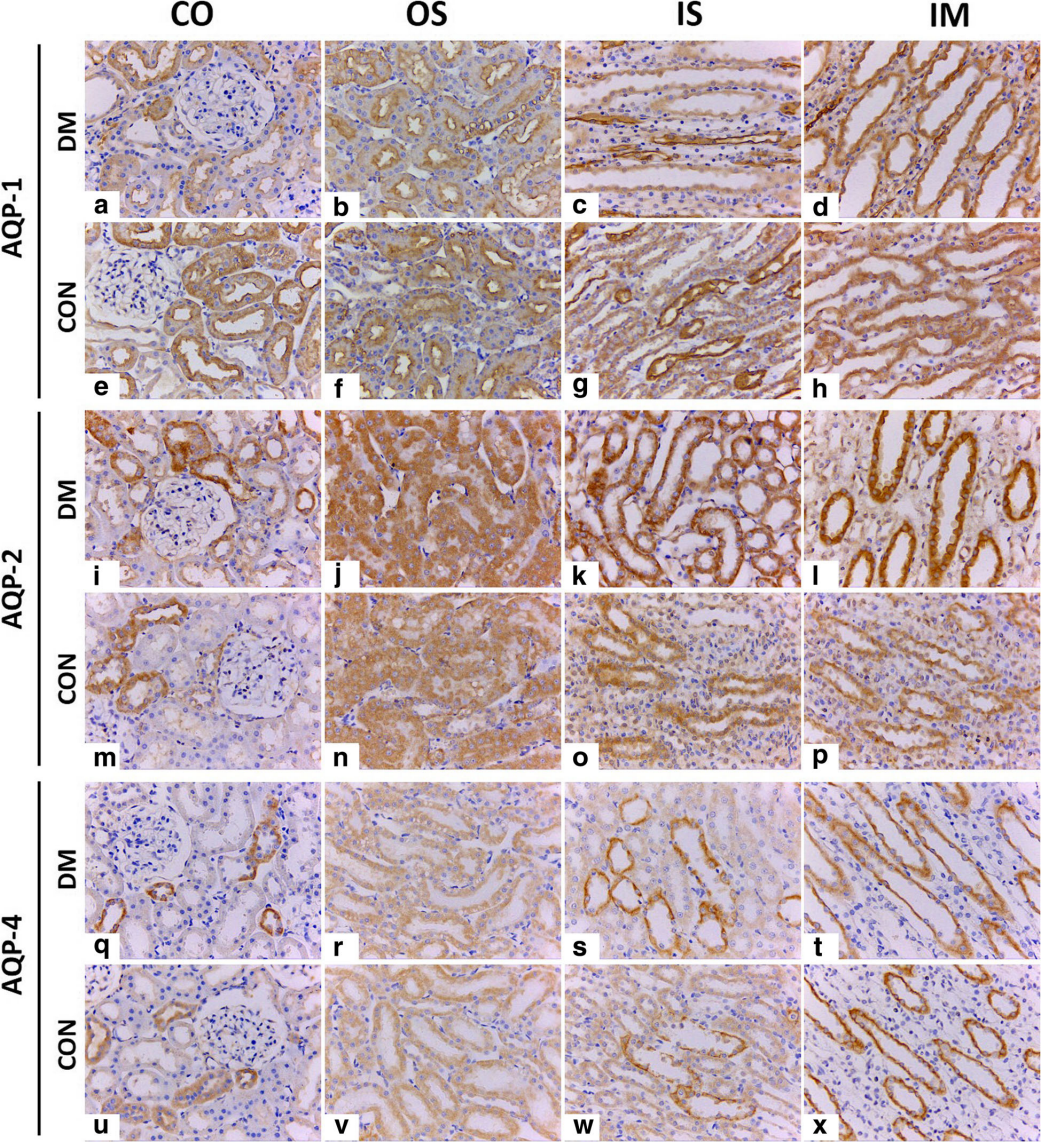
Immunocytochemical analysis of aquaporins (AQPs) on four different anatomical layers of the kidney. No difference was found between the group of streptozotocin-induced diabete mellitus (DM) animals (**a**-**d**) and the control group (**e**-**h**) in their AQP-1 expression on different anatomical layers of the kidney. Labeled AQP-1 was observed in the proximal tubules and Henle’s loops. The most intense labeling was found in the cortex and in the outer stripe of the outer medulla (**a**, **b** and **e**, **f**) in both groups. Labeled AQP-2 was observed on the apical plasma membrane and in intracellular cytoplasm domains. The labeling was the most intense in the outer stripe of the outer medulla (**j** and **n**). There was a noticeable increase in the AQP-2 labeling in OS, IS and IM in DM animals (**j**-**l**) compared with control animals (**n**-**p**). There was no significant difference between the tow group in there AQP-2 expression in CO (**i** and **m**). There was no significant difference between DM group (**q**-**t**) and control group (**u**-**x**) in their AQP-4 expression on different anatomical layers of the kidney. AQP-4 was detected in the collecting ducts and the proximal tubules. AQP-4 labeling was most intense in the inner medulla (**t** and **x**). Magnification × 400

In both DM and control kidney sections were found AQP-1, AQP-2 and AQP-4 immunoreactive cells (Fig. 2). AQP-1 staining and AQP-2 staining were prominent and widespread in different anatomical layers of the kidney in the diabetic rats. The OD value of AQP-2 expression in the OS, IS and IM in diabetic animals was significantly higher than that in controls (Table 3). Although the OD value of AQP-2 expression in CO in the DM animals was significantly higher than that in controls at *p* < 0.050 level, there was no significant difference after Bonferroni’s correction. However, there was no significant difference between the two groups in their OD values of AQP-1 and AQP-4 expression (Table 3).

### Correlation between ADC values and AQP-2

**Fig. 3:**
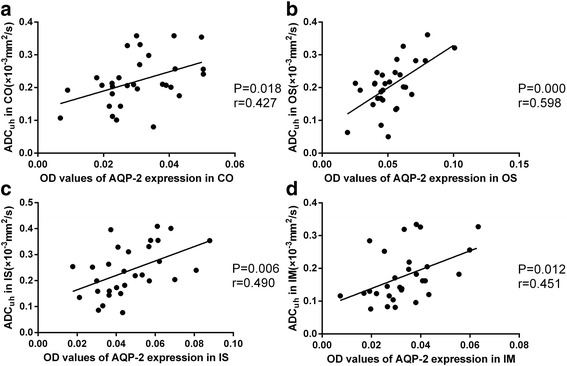
Correlation between renal optical density (OD) values of aquaporin-2 (AQP-2) expression and values of apparent diffusion coefficient obtained with ultra-high b-value (ADC_uh_). Significantly positive correlations are depicted for the outer stripe of the outer medulla in (**b**) (*r* = 0.598, *p* < 0.001), the inner stripe of the outer medulla in (**c**) (*r* = 0.490, *p* = 0.006,) and the inner medulla in (**d**) (*r* = 0.451, *p* = 0.012). Although there was a positive correlation between ADCuh and OD values of AQP-2 expression in cortex in (**a**) (*r* = 0.427, *p* = 0.018), there was no significant difference after Bonferroni's correction

ADC_uh_ values were positively correlated with OD values of AQP-2 expression in the OS, IS and IM of the kidney (Table 4, Fig. 3). Although there were positive correlations between ADC_uh_ and OD values of AQP-2 expression in CO at the *p* < 0.050 level, there was no significant difference after Bonferroni’s correction. The ADC_low_ and ADC_st_ values on the four anatomical layers of the kidney were not correlated with the OD values of AQP-2 expression. In addition, there were no correlations between ADCs and OD values of AQP-1 and AQP-4 expression (Table 4).

## Discussion

We used an STZ-induced model of diabetes which used a high-fat diet combined with a low STZ dose. The animals developed features of type-1 and type-2 DM with reduced insulin production and symptoms of a metabolic syndrome as previously described [12]. All the high-fat-diet and STZ-treated rats developed typical hyperglycaemia, increased urine output, and loss of body weight as typical symptoms of diabetes. In our diabetic model of animals, serum urea increased significantly, which is consistent with previous studies [7].

During the progression of diabetic nephropathy, the primary pathological changes involved fibrosis, impaired renal function, as well as chronic kidney diseases, all of which are known to be associated with a decrease in ADC both in human and in animal studies [13–15]. However, in our study, ADC_low_ and ADC_st_ of DM animals and control animals did not show any significant change. These results are in accordance with those of Cakmak et al. [5], who reported no significant difference in ADC values between the control group and earlier diabetic stages (stage 1 and stage 2). In an experimental rat diabetic nephropathy study by Ries et al. [6], no statistically significant difference was found between CO, IS and IM except for a subgroup of diabetic animals with oedematous cellular damage. In a study by Hueper et al. [7], the ADC values of the DM animals and controls were not different in their medulla and cortex. Interestingly, they found that medullary and cortical ADC was even elevated in diabetic animals with unilateral nephrectomy, which they explained by using low b-values of 0 and 300 s/mm^2^. However, if ADC_low_ values were sensitive enough to the perfusion change within the kidney, higher ADC_low_ values (due to hyperfiltration in the diabetic kidneys) should have been found in our study. The increased ADC in DM with unilateral nephrectomy in the study by Hueper et al. [7] was caused not only by hyperfiltration but also by a compensatively increased renal blood flow due to unilateral nephrectomy. Therefore, our results indicated that ADC_low_ and ADC_st_ measurements seem to reflect histopathological changes within the tissue not sensitive to the energy metabolism or the tubular flow or reabsorption.

We further suggest that ADC_uh_ may serve as a promising biometric for the early detection of diabetic nephropathy. Moreover, ADC_uh_ was lower than the ADC_st_ and ADC_low_ values, which corresponds with the finding that the higher b-value in the scan, the lower the estimated ADC values in the kidney [16]. ADC contained information not only about diffusion but also about perfusion and flow effects. Some studies [17] suggested that diffusion signal intensity with low b-values is mainly contributed by a fast diffusion component, as ADC_low_ is mainly illustrated by the fast blood perfusion in the kidney. Diffusion signal intensity with high b-values is dominated primarily by a slow diffusion component, as ADC_st_ is related to the slow water diffusion. Thus, according to the relationship between the b-value and diffusion signal intensity, the ADC_uh_ measured at ultra-high b-value might have something to do with a slower diffusion component because we observed a weak ADC_uh_ in the present study. Moreover, our findings indicate that ADC_uh_ measurement is more sensitive than ADC_low_ and ADC_st_ measurement to the energy metabolism or the tubular flow or reabsorption.

The changes we found in ADC_uh_ may correlate with the changes in AQP expression. In this research we used immunohistochemistry to measure the expression of AQP-1, AQP-2 and AQP-4 and we found that OD values of AQP-2 expression increased in the OS, IS and IM whereas expression of AQP-1and AQP-4 in different anatomical layers of the kidney in the diabetic animals did not change compared with controls. Aquaporins play an important role in the reabsorption of water from renal tubular fluid. In one study, polyuria and the inability to concentrate their urine has been reported in AQP-1-deficient mice [3]. In long-term type-1 diabetic rats, polyuria probably results from a reduced AQP-1 expression in renal tissues [18]. Earlier studies using STZ-induced animal models of DM also reported an increased AQP-2 expression in the kidneys [19–23]. Our finding agrees with the abovementioned results and supports the proposition that an increased AQP-2 expression might work as a compensatory mechanism that alleviates dehydration.

In recent studies, attention has been paid to the relationship between the ADC and AQP expression in the membranes. A clinical study using ultra-high b-value-DWI in Parkinson’s disease suggests that ADC_uh_ may help to illustrate the function of the AQP [9]. In addition, the speed of water transportation by AQPs in the membrane is about 0.45 × 10^-3^ mm/s slower than the water diffusion speed between cell gaps [24]. Moreover, our study showed a positive correlation between ADC_uh_ and AQP-2 expression, even though only a limited correlation. Thus, based upon these findings, we propose that DWI-MRI with ultra-high b-values might be able to assess water transportation by AQPs.

There are several limitations in the present study. First, we only measured at one time point during the progression of the disease. Second, the investigated animal population was relatively small. A large number of subjects and multiple time point measurements during the progression of the disease can help to confirm our findings. Third, the higher b-values we used may lead to a lower signal-to-noise ratio. However, we used a 7.0-T small-animal scanner to compensate the drawback, which may help us obtain a more powerful signal-to-noise ratio. Another limitation is that the regions of interest were drawn manually. Finally, as optical OD values of AQP expression were semiquantitative they may not be highly accurate because OD values are susceptible to the condition of staining and exposure duration when the images are taken. However, this drawback may be compensated by the immunohistochemistry that we used to achieve OD values, which were stained under the same condition and in the same batch. Images were taken at the same exposure duration to make sure that our data are true and comparable.

In conclusion, we found significantly higher ADC_uh_ values in CO, OS and IS and AQP-2 expression in the OS, IS and IM in DM animals compared with controls. Thus, we suggest that AQP-2 plays an important role in alleviating dehydration caused by poorly uncontrolled diabetes with polyuria and that DWI with ultra-high b-values may be valuable for noninvasive early detection and discriminative diagnosis of diabetic nephropathy. Lastly, the change in ADC_uh_ may reflect function of the AQP.

## Abbreviations

ADC: Apparent diffusion coefficient
ADC_low_: ADC calculated using low b-values
ADC_st_: ADC calculated using standard b-values
ADC_uh_: ADC calculated using ultra-high b-values
AQP: Aquaporin
CO: Cortex
DM: Diabetes mellitus
DWI: Diffusion-weighted imaging
IM: Inner medulla
IS: Inner stripe of the outer medulla
OD: Optical density
OS: Outer stripe of the outer medulla
STZ: Streptozotocin
